# Prevention of human Abeta induced synaptic deficits by hAPOE4

**DOI:** 10.1002/alz70855_106907

**Published:** 2025-12-24

**Authors:** Angela Gomez‐Arboledas, Enikö Kramár, Shimako Kawauchi, Celia Da Cunha, Yueh Hao Lu, Giedre Milinkeviciute, Ian F Smith, Marcelo A Wood, Andrea J Tenner, Frank M. La Ferla, Grant R MacGregor, Kim N. Green

**Affiliations:** ^1^ University of California, Irvine, Irvine, CA, USA; ^2^ University of California Irvine, Irvine, CA, USA; ^3^ University of California Irvine, IRVINE, CA, USA

## Abstract

**Background:**

Genome‐Wide Association Studies (GWAS) identified ApoE4 as the strongest genetic risk factor for late‐onset Alzheimer's Disease (LOAD). As part of our efforts to develop mouse models that better recapitulate LOAD, at Model Organism Development & Evaluation for Late‐Onset Alzheimer's Disease (MODEL‐AD) consortium at University of California – Irvine, we have created a triple homozygous mouse model that combines our previously developed hAb‐KI^loxP^ mice (Jackson Lab #031050), a humanized ApoE4, which replaces part of the murine ApoE locus, (Jackson Lab #027894) and a humanized MAPT‐GR (H2). This newly developed mouse model (MAD1; Jackson Lab #038104) will allow us to evaluate the interactions between aging, hAPOE4, hTau and hAb.

**Method:**

Mice were aged to 4, 12, 18 and 24 months of age (hAb‐KI^loxP^ HO;hApoE4 HO cohort) or 4 and 12 months of age (MAD1 cohort). At these timepoints, coronal hippocampal slices were prepared, and long‐term potentiation recordings were obtained. Synaptic density and microglial synaptic engulfment were also assessed by super‐resolution microscopy in different brain regions.

**Result:**

hAb‐KI^loxP^ mice showed a significant reduction in mean potentiation 50‐60 minutes post TBS, when compared to WT mice, indicative of an LTP deficit associated to human Ab. Interestingly, the presence of hAPOE4 rescued the LTP deficits observed on the hAb‐KI^loxP^ mice from 4 months of age. Additionally, we observed a significant pre‐synaptic loss on the hAb‐KI^loxP^ mice and hAb‐KI^loxP^ HO;hMAPT HO (vs WT) that was prevented by hAPOE4. Interestingly, this pre‐synaptic loss correlated with increased microglial synaptic pruning in the hAb‐KI^loxP^ mice, which was then reduced by the presence of hAPOE4.

**Conclusion:**

hAb induces robust LTP deficits that are prevent by hAPOE4, from 4 months of age. These results are in agreement with the excessive synaptic loss observed in the hAb‐KI^loxP^ mice, which is also rescued by the addition of the hAPOE4 variant. Further studies are needed to fully understand the differences between murine and human APOE4 and how this could be modulating the effects of hAb on synaptic integrity and function.

